# Convergent Evolution of Chromosomal Sex-Determining Regions in the Animal and Fungal Kingdoms

**DOI:** 10.1371/journal.pbio.0020384

**Published:** 2004-11-09

**Authors:** James A Fraser, Stephanie Diezmann, Ryan L Subaran, Andria Allen, Klaus B Lengeler, Fred S Dietrich, Joseph Heitman

**Affiliations:** **1**Department of Molecular Genetics and Microbiology, Duke University Medical CenterDurham, North CarolinaUnited States of America; **2**Howard Hughes Medical Institute, Duke University Medical CenterDurham, North CarolinaUnited States of America; **3**Duke Institute for Genomics Sciences and Policy, Duke University Medical CenterDurham, North CarolinaUnited States of America; **4**Department of Medicine, Duke University Medical CenterDurham, North CarolinaUnited States of America; **5**Department of Pharmacology and Cancer Biology, Duke University Medical CenterDurham, North CarolinaUnited States of America

## Abstract

Sexual identity is governed by sex chromosomes in plants and animals, and by mating type (MAT) loci in fungi. Comparative analysis of the MAT locus from a species cluster of the human fungal pathogen *Cryptococcus* revealed sequential evolutionary events that fashioned this large, highly unusual region. We hypothesize that MAT evolved via four main steps, beginning with acquisition of genes into two unlinked sex-determining regions, forming independent gene clusters that then fused via chromosomal translocation. A transitional tripolar intermediate state then converted to a bipolar system via gene conversion or recombination between the linked and unlinked sex-determining regions. MAT was subsequently subjected to intra- and interallelic gene conversion and inversions that suppress recombination. These events resemble those that shaped mammalian sex chromosomes, illustrating convergent evolution in sex-determining structures in the animal and fungal kingdoms.

## Abstract 2

A comparative genomic analysis of the sex determining region in fungi reveals a remarkable similarity between its evolution and the events which shaped mammalian sex chromosomes

## Introduction

**Figure 1:**
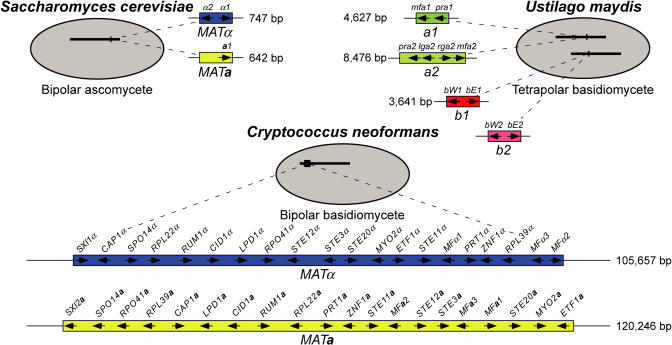
Fungal *MAT* Locus Paradigms Interaction of mating partners during the fungal sexual cycle is directed by bipolar or tetrapolar mating systems. The budding yeast S. cerevisiae is an ascomycete with bipolar mating (graphic at upper left). The maize pathogen U. maydis is a tetrapolar basidiomycete with multiple mating types conferred by two mating type loci (graphic at upper right). One *(a)* is biallelic and encodes pheromones and pheromone receptors, while the second *(b)* is multiallelic and encodes homeodomain transcription factors. In contrast, the human pathogen C. neoformans (lower graphic) is a basidiomycete with a bipolar system with only two mating types (**a** and α). The C. neoformans MAT locus encodes homeodomain transcription factors, pheromones and pheromone receptors, other elements of the pheromone activated MAPK cascade, and many genes whose role in mating, if any, is at present unknown.

Elucidating mechanisms by which sex chromosomes evolved from autosomes has been accelerated by the revolution in genomic sciences. In humans, the male-specific approximately 50–60 Mb Y chromosome evolved via chromosomal rearrangement, gene conversion, duplication, and degeneration over approximately 300 million years to give rise to four distinct evolutionary temporal groupings, or strata (Lahn and Page 1999; Skaletsky et al. 2003). In contrast, fungi have much less extensive sexually dimorphic chromosomal regions; in the budding yeast *Saccharomyces cerevisiae,* the **a** and α mating types are established by the mating type (MAT) locus, which spans only 642 bp or 747 bp, respectively, and encodes only one or two cell type factors (Figure 1) (Herskowitz 1989). Recent studies of the evolution of ascomycete MAT loci have shown that, despite significant changes in both content and structure, small size has remained a common feature (Tsong et al. 2003; Butler et al. 2004). In contrast to mammals and other obligate diploid organisms, fungi are viable as both haploids (the equivalent of gametes in other systems) and diploids. This has influenced the evolution of genes in the sex-determining regions in the two systems, as it enables those in obligate diploids to degenerate to nonfunctional alleles in one of two sex-determining chromosomes.

Unlike the exclusively bipolar mating in ascomycetes, basidiomycete fungi usually have more complex tetrapolar mating, in which two unlinked genomic regions establish cell identity, and both must differ for sexual reproduction (Kahmann et al. 1995; Kronstad and Staben 1997; Casselton and Olesnicky 1998). One locus encodes pheromones and pheromone receptors, while the second encodes homeodomain transcription factors (Figure 1). However, like *S. cerevisiae,* some haploid basidiomycete species, such as the human fungal pathogen *Cryptococcus* *neoformans,* exhibit bipolar mating, in which a single locus establishes mating type*.* Unlike S. cerevisiae and *Schizosaccharomyc pombe,* which are homothallic fungi that switch mating type via recombination between silent and active MAT cassettes, *Cryptococcus* is a heterothallic fungus that has never been observed to switch mating type and lacks any silent MAT cassettes. In contrast to the more restricted ascomycete MAT loci, the C. neoformans MAT locus is unusually large (spanning over 100 kb) and contains more than 20 genes (Figure 1) (Lengeler et al. 2002). Previous studies of this region of the genome have shown the MAT region to be recombinationally suppressed, with meiotic segregants from a cross each receiving a single intact, nonrecombined locus of either the MAT**a** or MATα type (Hull and Heitman 2002; Lengeler et al. 2002). The MAT locus orchestrates sexual development involving cell fusion, formation of dikaryotic hyphae, and subsequent nuclear fusion, meiosis, and sporulation to produce the suspected infectious particles. The α allele of the C. neoformans MAT locus has been linked to environmental prevalence, virulence, differentiation capacity, and unusual fecundity in a recent outbreak (Kwon-Chung et al. 1992; Wickes et al. 1996; Fraser et al. 2003).

**Figure 2:**
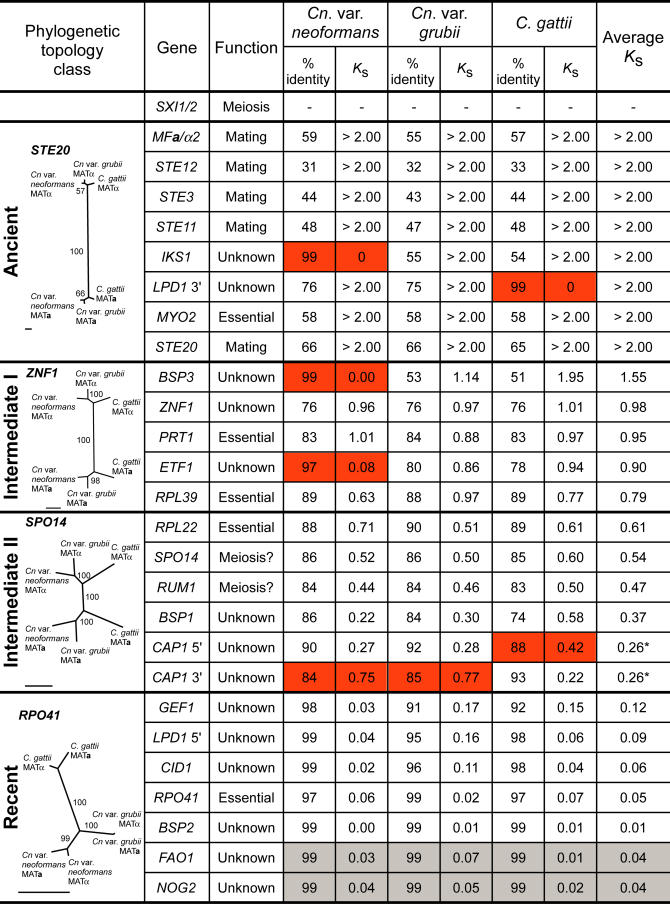
Genes of the MAT Locus Comparison of **a** and α alleles of the MAT genes in the three *Cryptococcus* lineages based on percent nucleotide identity between the coding sequences of the **a** and α alleles within that species. *K_s_* values were calculated from comparison of the **a** and α alleles in each species. Genes with unusual *K_s_* values are shown in red, and genes from regions flanking MAT are shown in grey. Phylogenetic trees are based on maximum likelihood analysis (scale bar = 0.05 substitutions per site), and are labeled with the phylogenetic class they represent. Further details are presented in Figures S1 and S2. *The Average *K*_s_ for *CAP1* was calculated after excluding the indicated unusual values.

C. neoformans exists as two subspecies—*Cn.* var. *grubii* and *Cn.* var. *neoformans—*that diverged approximately 20 million years ago (mya), and the MAT locus has been characterized in both (Xu et al. 2000; Lengeler et al. 2002). The C. neoformans MAT locus encodes the pheromones, pheromone receptors, and homeodomain factors that are usually distributed between the two tetrapolar loci in this phylum, as well as additional pheromone response pathway elements and proteins from many other functional categories (Lengeler et al. 2002), including essential genes (Figure 2). As in the multicellular eukaryotes, this sex-determining structure is large; the impending completion of the *Cn.* var. *neoformans* genome reveals that the MAT locus occupies 6% of a 1.8-Mb chromosome in a genome of approximately 20 Mb. Analogous to the human Y chromosome, the sex-determining genes are scattered among others seemingly unrelated to sex. Here we show, on the basis of comparative genomic analysis using new sequences isolated from a primary pathogenic sibling species *Cryptococcus* *gattii,* that sequential evolutionary events that fashioned this large, highly unusual region of the *Cryptococcus* genome can be reconstructed. The *Cryptococcus* MAT locus therefore provides insights into how complex, dimorphic sex-determining regions evolved from simpler loci containing only one or two genes.

## Results

The closest known relative to C. neoformans is the sibling species C. gattii, a primary human pathogen which diverged approximately 40 mya (Xu et al. 2000, Kwon-Chung et al. 2002). C. gattii therefore provides a unique vantage point from which to analyze MAT evolution, via comparative genomics, from a species cluster of human fungal pathogens.

**Figure 3:**
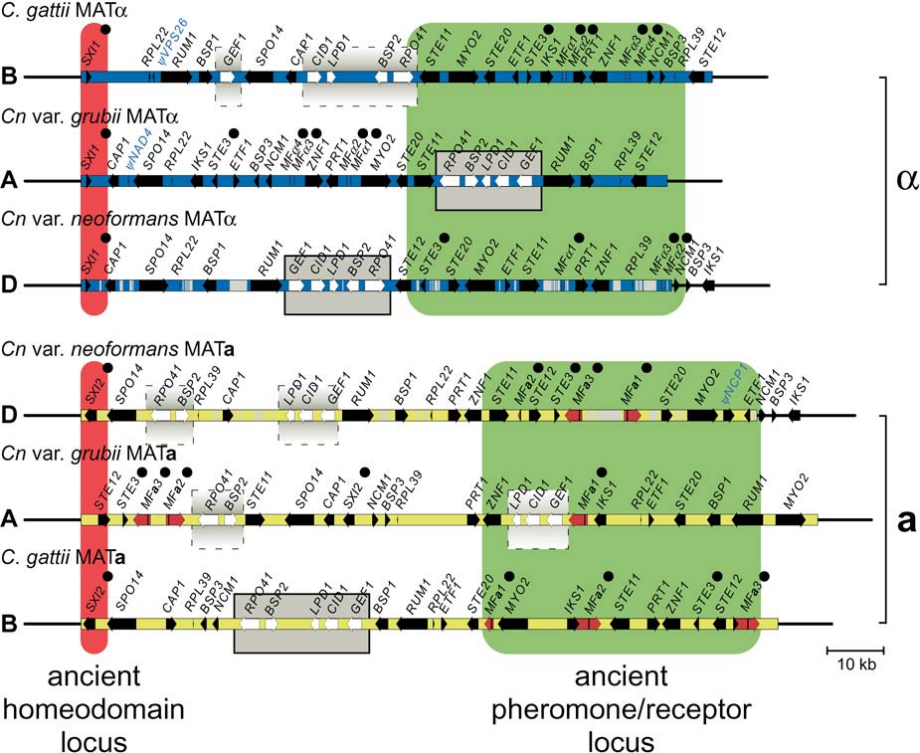
The Structure of MAT Is Highly Rearranged, with Divergent Gene Alleles Embedded in Syntenic Genomic Regions The nonrecombining α (blue) and **a** (yellow) MAT alleles from the divergent but related species are depicted, spanning more than 100–130 kb and including 10 kb of common flank regions on the left and right demarcated by sharp borders with MAT. The original locations of ancient tetrapolar loci proposed to have given rise to MAT are shown in red (ancestral homeodomain locus) and green (ancestral pheromone/receptor locus), with the most ancient genes (encoding homeodomain transcription factors, pheromones and pheromone receptors) bulleted. Genes that show mating type-specific phylogeny are shown in black, and genes with species-specific phylogeny are white. Synteny between the genes with species-specific phylogeny is indicated with grey boxes. Pseudogenes are labeled in blue, and grey bars represent repeated elements in *Cn.* var. *neoformans*. Red arrows represent pheromone amplicons.

**Figure 4:**
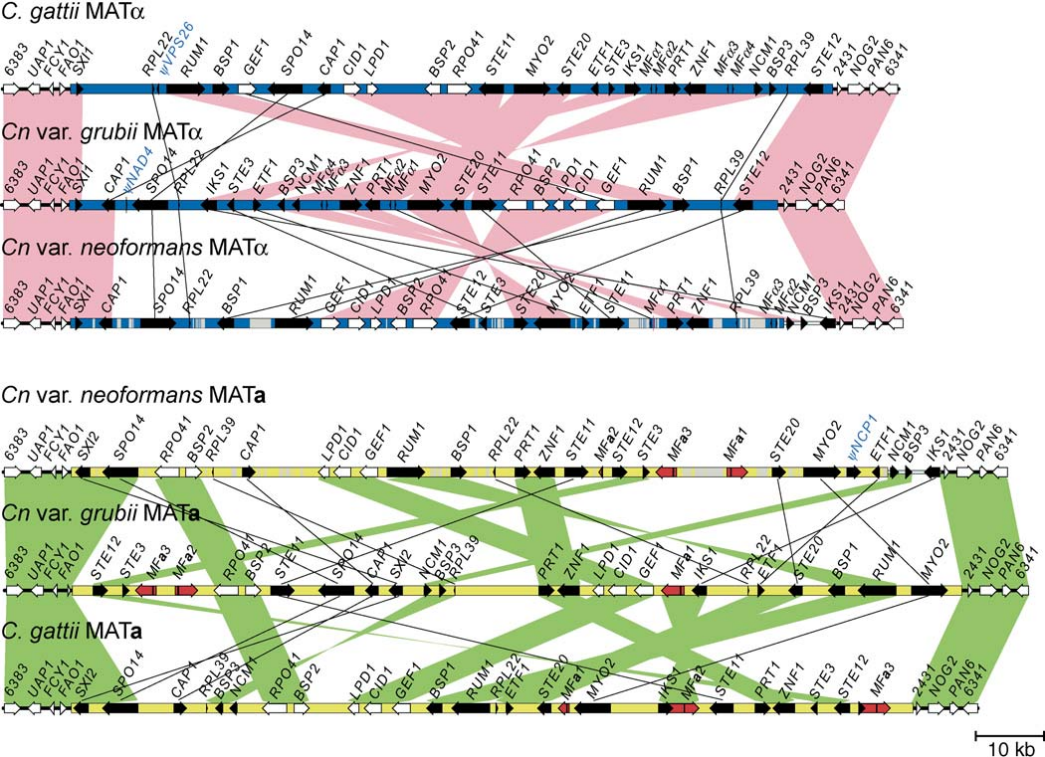
MAT Is Highly Rearranged between Species and Mating Types The genomic region spanning the nonrecombining α (blue) and **a** (yellow) MAT alleles from the divergent but related species is depicted, with pink and green colored bars representing regions of synteny, and black lines the relative positions of genes whose position is not conserved. Black arrows depict mating type-specific genes. White arrows represent genes with a species-specific phylogeny. Red arrows represent pheromone amplicons.

The **a** and α alleles of the MAT locus were cloned and sequenced from two representative C. gattii strains (AY10429, AY10430). Figure 3 shows the structures of the six known MAT alleles. Four features are prominent. First, the mating type-specific sequences span more than 100 kb in all six alleles. Second, the MAT-specific sequences are separated from the genome by sharply demarcated borders; the flanking regions share over 99% nucleotide sequence identity and syntenic gene order, whereas the sequences within MAT are divergent (Figures 4 and S1). Third, comparison of the MAT alleles reveals few genes unique to one mating type (encoding factors Sxi1α and Sxi2**a**, the only MAT homeodomain proteins) with the locus composed almost entirely of divergent alleles of a common gene set. Fourth, the MAT gene cohort has been dramatically rearranged during evolution (Figure 4). Whole-genome analysis of *Cn.* var. *neoformans* at Stanford and The Institute for Genomic Research (TIGR), *Cn.* var. *grubii* at Duke and the Broad Institute, and C. gattii at the University of British Columbia and the Broad Institute, reveal that these rearrangements at MAT are highly atypical compared to the non-MAT regions of the genome in all three species (B. J. Loftus, unpublished data; J. W. Kronstad, personal communication). In addition to the original set of shared genes (Lengeler et al. 2002), comparative analysis employing the new C. gattii sequences revealed that an additional five novel genes are present in all six characterized alleles, including one predicted noncoding gene with no apparent open reading frame. All five were confirmed by RT-PCR analysis to be expressed (unpublished data). In total, genic sequences comprise approximately 50% of MAT (see Figure 3).

Our model for the evolution of this unusual structure is that two unlinked sex-determining regions of the genome expanded by acquiring genes of related function, and these two novel gene clusters were then captured into a common genomic region by a chromosomal translocation, entrapping still further genes. This resulted in a tripolar transitional intermediate mating system that collapsed via gene conversion to result in the contiguous, linked MAT alleles and a bipolar system. MAT was then subject to inversions that suppressed recombination, punctuated by ongoing rounds of inter- and intra-allelic gene conversion. Below, we summarize the analyses that support this model.

**Figure 5:**
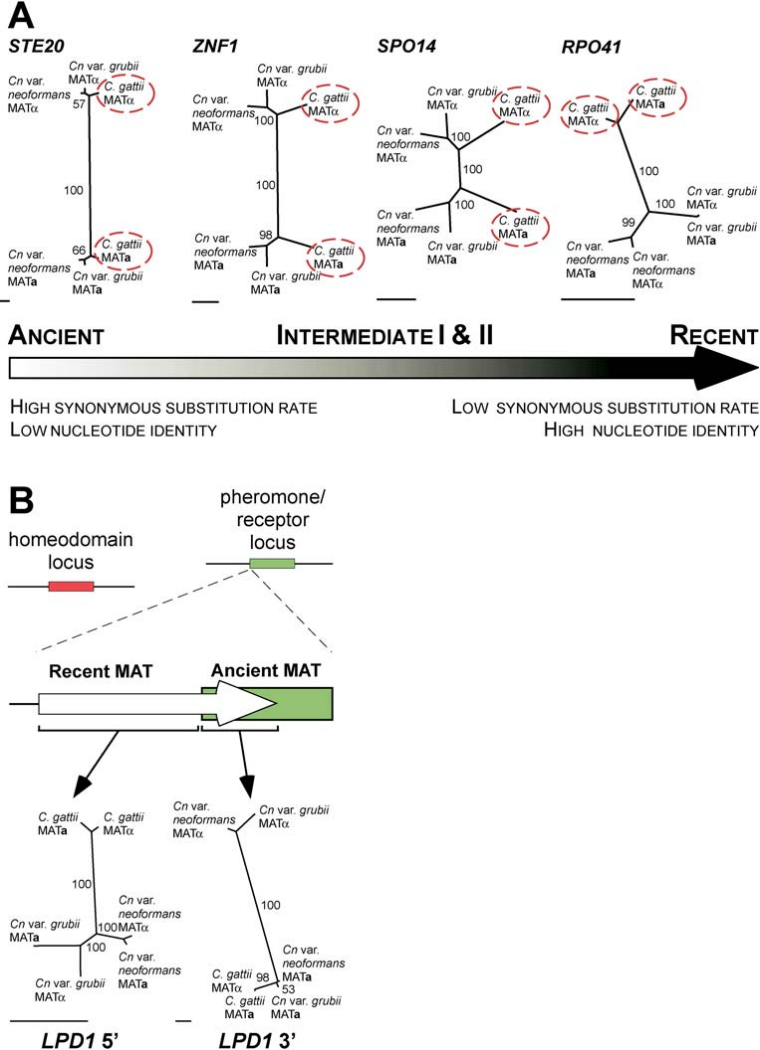
MAT Genes Have Different Phylogenetic Histories (A) The genes of the MAT locus can be separated into four distinct groupings based on phylogenetic class, synonymous substitution rate, and nucleotide identity. The C. gattii alleles in each phylogram are encircled in red. (B) The *LPD1* gene defines an ancient border of MAT. The 5′ end of the coding region is species-specific, while the 3′ region is mating type-specific. The ancient homeodomain locus is shown in red, and the ancient pheromone/pheromone receptor locus in green. Maximum likelihood trees are shown. Scale bar represents 0.05 substitutions per site. Further details are provided in Figures S2 and S3.

Both maximum likelihood and parsimony analyses reveal that MAT is constructed from genes with different phylogenetic histories; comparing the topologies of the phylograms for each protein-coding gene showed four major classes (Figures 5A, S2, and S3). The first class, which we call “ancient,” contains genes in which the alleles share a low level of nucleotide identity and cluster into distinct **a** and α clades. This pattern represents the most ancient genes contained within this recombinationally suppressed region of the genome, and includes those encoding pheromones, pheromone receptors, and elements of the pheromone-sensing mitogen-activated protein kinase (MAPK) pathway. The second and third classes (which we term “intermediate I” and “intermediate II,” respectively) represent intermediate genes that have progressively higher nucleotide identity between the **a** and α alleles, and less discrete, although still MAT-specific, phylogenetic patterns (Figures 2 and 5A). This pattern reflects genes that have been contained within this recombinationally suppressed region for shorter periods of time. Finally, the last group (“recent”) comprises the five most recently acquired genes that exhibit a species-specific, but not MAT-specific, phylogenetic pattern, similar to genes outside MAT (see Figure 2). These distinct patterns mirror the relative length of time each gene has spent within the largely nonrecombining MAT locus (ranging from ancient to recent acquisitions), and provide insight into how this large genomic structure was fashioned. Stated differently, the divergence times for the ancient and intermediate classes in C. gattii and C. neoformans are equivalent (represented by *STE20,* *ZNF1,* and *SPO14* in Figure 5), as these genes entered MAT prior to speciation, while the recent class (represented by *RPO41*) began diverging after this time. The tree topologies of *STE20, ZNF1, SPO14,* and *RPO41* were shown to be statistically different (*p* < 0.0001) by the Shimodaira-Hasegawa test (Shimodaira and Hasegawa 1999).

All six MAT alleles contain the same set of five genes that exhibit the unusual species-specific phylogenetic pattern, suggesting that each allele has recruited the same cohort of genes by a common mechanism. One hypothesis we initially considered to explain acquisition of a common gene set is that the locus expanded by recruiting flanking genes. The *IKS1* gene is an integral component of the MAT locus in two lineages *(Cn.* var. *grubii* and *C.* *gattii),* but is a flanking gene in the third *(Cn.* var. *neoformans),* providing a unique opportunity to test this model (see Figure 4). Phylogenetic analysis reveals that the *IKS1* gene tree resembles phylograms for an ancient MAT-specific gene, with the exception of the *Cn.* var. *neoformans* lineage, in which gene conversion has fixed the α allele in both mating types with concomitant loss of the ancestral **a**-specific allele (Figures S2 and S3). The *ETF1, BSP3,* and *NCM1* genes share a similar evolutionary history. These genes are therefore not recently acquired, and instead provide examples of gene eviction from MAT by interallelic recombination to result in a reduction in MAT size and complexity in the *Cn.* var. *neoformans* lineage.

An alternative, more parsimonious model to explain why an identical set of five species-specific genes is present in all six MAT alleles is that these genes were acquired once in the progenitor MAT locus. However, their high level of synteny suggests recent acquisition; the *GEF1, CID1,* and *LPD1* genes are clustered in five of the six MAT alleles, the *BSP2* and *RPO41* genes are adjacent in all six, and synteny of the entire five-gene cluster has been maintained in three alleles (see Figure 3). Our model is that the two ancestral unlinked sex-determining regions were juxtaposed by a chromosomal translocation, entrapping this set of recently acquired common genes which were then subjected to more recent gene conversion.

An indication of the relative time over which alleles have resided within MAT and diverged can be inferred from the rate of synonymous mutations *(K_s_),* which accumulate over time and are nearly neutral with respect to selection in organisms such as *Cryptococcus,* in which strong codon bias is absent (Li 1993; Lahn and Page 1999; Nakamura et al. 2000). If we compare the alleles of genes outside MAT from **a** and α strains of the same species, their *K_s_* values are close to zero, reflecting freely recombining regions of the genome (see Figure 2). By contrast, genes embedded in MAT are largely nonrecombining, and therefore have *K_s_* values based on their divergence since the time of acquisition to the locus. The higher the *K_s_* value, the longer the genes have been diverging due to entrapment in MAT. This analysis reveals four major classes of genes, which correspond to the ancient (*K_s_* > 2.00), intermediate class I (2.00 > *K_s_* > 0.70), intermediate class II (0.60 > *K_s_* > 0.35), and most recently acquired genes (*K_s_* < 0.17) (see Figure 2). These gene classes are analogous to those shown by phylogenetic analysis. The MAT locus genes therefore partition into four primary groupings based on phylogeny, nucleotide identity, and synonymous substitution rates.

**Figure 6:**
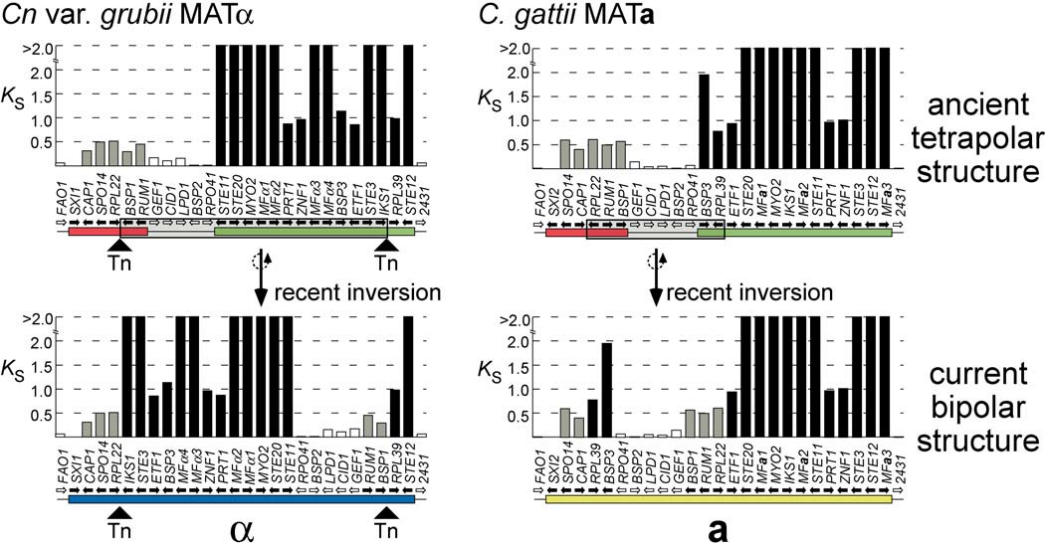
Reconstructing the Ancient MAT Alleles by Inversion-Mediated Rearrangement Plotting the synonymous mutation rate *(K_s_)* of each protein coding gene in MAT reveals that the different classes of genes in the two least rearranged loci (*Cn.* var. *grubii* MATα and C. gattii MAT**a**; see Figure 4) can be clustered by a single inversion. This may represent an ancient linked tetrapolar system—one cluster contains the pheromone and pheromone receptor genes (green bars), and the other a homeodomain-encoding gene (red bar). Transposon remnants are present at the extrapolated inversion breakpoint regions in *Cn.* var. *grubii*, as indicated (Tn). *K_s_* cannot be calculated between the *SXI1α* and *SXI2**a*** genes, because these are unrelated and not alleles, in contrast to other genes in the locus.

In the human Y chromosome, analysis of substitution rates reveals four temporal clusters, or strata, representing the sequential acquisition of genes to the male-specific region (Lahn and Page 1999). The genes in the *Cryptococcus* MAT locus are no longer stratified by age, because the members of the four identified classes have been heavily shuffled during speciation and mating type divergence, and this rearrangement appears ongoing. However, an analysis of relative gene locations in MAT reveals two alleles in which higher levels of synteny are evident: *Cn.* var. *grubii* MATα and C. gattii MAT**a** (see Figure 4). The *K_s_* values of genes distributed in these alleles were analyzed to reconstruct the genomic architecture of the common ancestral structure. In both cases, genes from each *K_s_*-defined class can be clustered via a single inversion, creating identical groupings in both the **a** and α alleles—and we hypothesize that this represents the genomic architecture of an ancestral MAT locus (Figure 6). Assuming the components of the ancient tetrapolar system are represented by the pheromone and pheromone receptor genes in one group and homeodomain-encoding genes in the other, the ancient pheromone/pheromone receptor cluster is further divisible into two groups based on *K_s_* values (Figures 2 and 6). This implies two major expansion events of this component. First, an ancient recruitment of genes including pheromone-sensing cascade components previously implicated in fertility (*K_s_* > 2.00), followed by more recent acquisition of the intermediate class I genes, whose role in mating has not yet been studied (2.00 > *K_s_* > 0.70). In constrast, the intermediate class II genes (0.60 > *K_s_* > 0.35) were recruited by expansion of the ancient tetrapolar homeodomain-containing locus. In this model, the expanding ancestral loci are separated by the five genes that exhibit species-specific phylogenies (Figure 6).

We hypothesize that the species-specific genes were then incorporated into the MAT locus by inversions that fused and rearranged the ancient tetrapolar structures. This model is supported by two of the species-specific genes, *LPD1* and *RPO41,* which exhibit an unusual hybrid phylogeny; although the majority of their coding region exhibits a species-specific phylogeny, the 3′ regions are mating type-specific. In two of the three lineages *(Cn.* var. *grubii* and *Cn.* var. *neoformans)* the *LPD1* gene exhibits this hybrid phylogeny, but in C. gattii the 5′ region in both mating types resembles the α alleles, and a *K_s_* value of zero for this region is indicative of recent gene conversion (Figures 2, 5B, S2, and S3). Analogous to the *Amelogenin* locus of primates, which spans an ancient pseudoautosomal boundary (Iwase et al. 2003), this phylogeny suggests that the five entrapped genes were integrated into MAT while the boundary genes were in a state of transition from species to mating type-specificity. Furthermore, it supports models in which these genes maintain species-specific phylogeny via gene conversion.

Rearrangement of MAT may be driven by recombination between transposable elements. In the *Cn.* var. *grubii* α allele, the breakpoints of the postulated inversion lie in intergenic regions that contain remnants of the Tcn760 mariner-type transposon (Figure 6) (Lengeler et al. 2002). The *Cn.* var. *neoformans* MAT locus is rich in transposable elements and remnants (17.3% α and 13.2% **a**; see Figure 1), a feature shared with the sex chromosomes of humans and mice (Waterston et al. 2002). Comparison with the completed *Cn.* var.*neoformans* genome sequence revealed that this represents a greater than 5-fold enrichment relative to the rest of the genome, when the transposon-rich presumptive centromeric regions are excluded. In *S. cerevisiae,* transposons and their remnants may be principal sites at which chromosomes rearrange in response to growth selection (Dunham et al. 2002), and transposons have been implicated as drivers of genome evolution in a number of eukaryotes, including humans (Kazazian 2004). Similarly, repeated elements may have driven stochastic MAT rearrangements to produce its current structure and efface the vestiges of the ancestral evolutionary strata, as successive random small inversions appear to be a major evolutionary mechanism shaping the locus. Although inversions have previously been implicated in transposing gene order during S. cerevisiae evolution (Seoighe et al. 2000), they have occurred at an unprecedented level in MAT while sparing adjacent regions (see Figure 4).

In the human Y chromosome, gene decay has been competing with ongoing gene acquisition and conservation (Skaletsky et al. 2003). In *Cryptococcus,* which is viable as either a haploid or diploid, suppression of meiotic recombination in MAT has not led to loss of any genes, with two minor exceptions. First, unique 5′ truncated pseudogenes exist in three of the alleles (*ΨNCP1* in *Cn.* var. *neoformans* MAT**a**, *ΨNAD4* in *Cn.* var. *grubii* MATα, and *ΨVPS26* in *C.* *gattii* MATα). Second, the number of pheromone genes varies between alleles. The **a** alleles contain three unlinked 130-bp *MF**a*** pheromone genes embedded in 900–5,000 bp amplicons identical within an allele but not between species. In contrast, the alleles contain three or four *MFα* pheromone genes embedded in approximately 500-bp conserved repeats, usually flanking the syntenic *PRT1/ZNF1* gene pair in inverted orientation. These repeats are likely maintained by intra-allelic gene conversion, ensuring maintenance of these important fertility genes in the absence of meiotic recombination. This is a striking difference to the MAT locus of S. cerevisiae, where gene conversion plays the unrelated and very distinct role of driving mating type switching (Strathern and Herskowitz 1979; Wu and Haber 1996; Haber et al. 2004). In the C. gattii MAT**a** allele one *MF**a*** gene repeat has expanded into the adjacent *IKS1* gene, duplicating the *IKS1* 3′ region in a second amplicon. In *Cn.* var. *neoformans*, gene conversion has duplicated a retrotransposon fragment adjacent to *MFα1* into the *MFα2* repeat, while the fourth pheromone gene has been replaced by a retroelement, representing the only clear example of gene loss within MAT. We note that our gene disruption studies reveal that the MAT locus contains five essential genes (see Figure 2), and their presence likely constrains MAT to only those rearrangements that ensure their retention.

## Discussion

Our studies reveal that the MAT locus of C. neoformans is strikingly divergent from that of the model budding yeast *S. cerevisiae,* other ascomyctes, and even related basidiomycetes. Whereas the budding yeast MAT locus is quite small, and encodes only one or two key cell fate determinants that are sequence unrelated, the C. neoformans MAT locus spans over 100 kb, contains more than 20 genes, and with the exception of the *SXI1α* and *SXI2**a*** genes, is otherwise composed of divergent alleles of a common gene set.

The similarity between the MAT locus of S. cerevisiae and that of C. neoformans is restricted to the presence of related homeodomain proteins, suggesting that these may represent the most ancient components of the ancestral MAT locus that was shared between the ascomycete and basidiomycete lineages. One of several unusual features that the C. neoformans MAT locus does not share with the more restricted S. cerevisiae counterpart is the presence of several predicted essential genes, which we have confirmed by gene disruption studies (see Figure 2). Given the evidence for rampant inversions and translocations in MAT, the presence of these essential genes, which are spaced throughout the locus, may have served as an evolutionary brake to ensure that large regions of the MAT locus were not lost in haploid recombinants produced by the sexual cycle. We note that essential genes are represented both in the expanded pheromone signaling and homeodomain clusters of the ancestral tetrapolar mating system and within the set of five newly acquired, entrapped genes. The presence of these essential genes embedded within each component of the MAT locus may have thereby contributed to the expansion of the locus from the much smaller MAT loci common in ascomycetes and other basidiomycetes. Another marked distinction is that C. neoformans is a heterothallic yeast that has never been observed to undergo mating type switching, whereas S. cerevisiae is a homothallic yeast in which Ho endonuclease-mediated cleavage effects mating type switching by promoting recombination between the active and silent MAT cassettes. We find no evidence for silent mating type cassettes in *C. neoformans,* consistent with its classification as a heterothallic fungus. Furthermore, recent studies (Butler et al. 2004) have revealed the acquisition of silent mating type cassettes and both Ho-independent and Ho-dependent switching is restricted to ascomyete fungal lineages related to S. cerevisiae and *Sc. pombe*.

**Figure 7:**
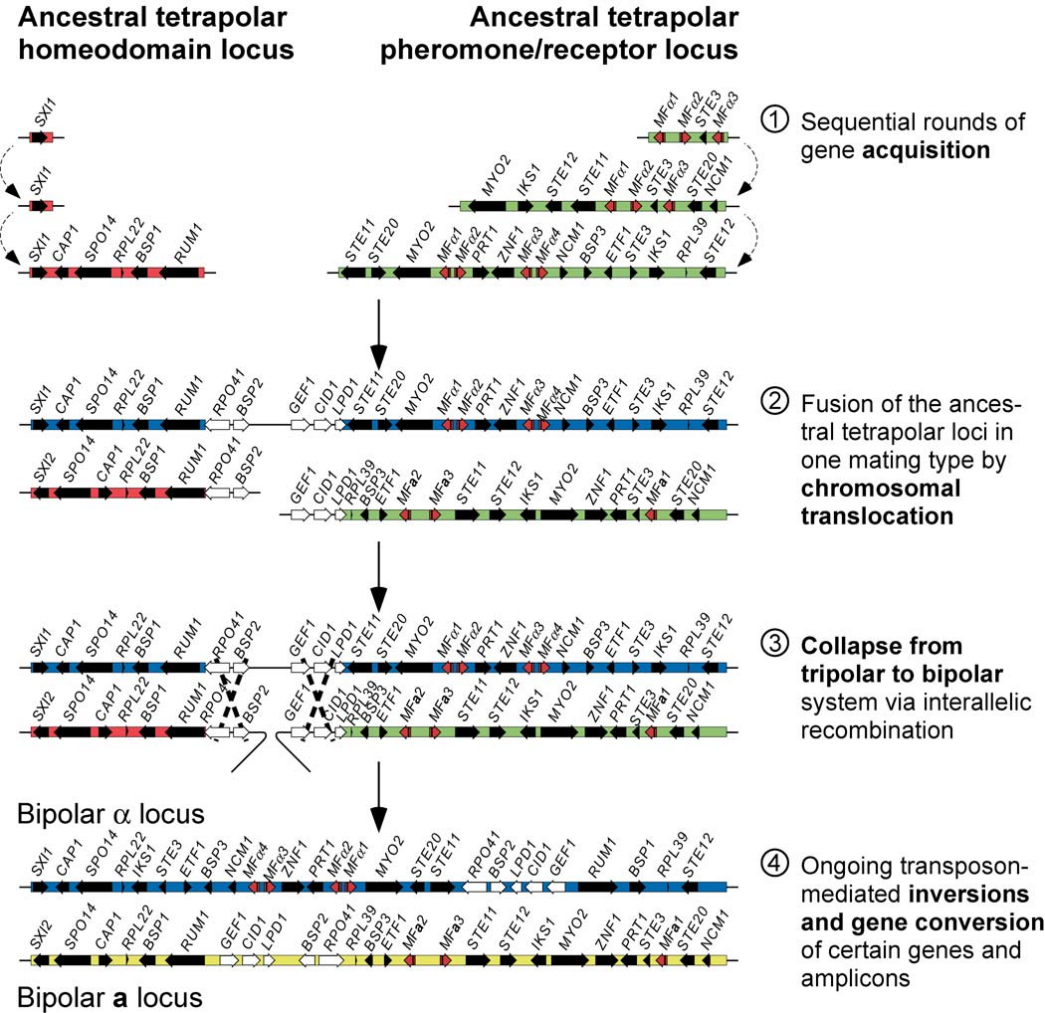
A Model for the Evolution of MAT Our evidence indicates that the ancient loci of a canonical tetrapolar system expanded to incorporate additional genes, beginning with two rounds of expansion of the pheromone/receptor locus: first to acquire genes including components of the pheromone-signaling MAPK cascade (ancient), and second to acquire genes whose role in mating is unknown (intermediate I). Next, the ancestral homeodomain locus acquired genes hypothesized to function in the dikaryon or meiosis (intermediate II). The tetrapolar loci in one mating type fused by chromosomal translocation, entrapping the most recently acquired species-specific gene set (recent) and creating a tripolar intermediate. A second locus fusion event then occurred, to link the two regions from the opposite mating type and create the bipolar ancestors of MAT. Subsequent inversion-mediated rearrangements have erased the discrete evolutionary strata.

We propose a model of MAT evolution that addresses the four distinct evolutionary classes in which the genesis of a bipolar system occurred in the progenitor of the three *Cryptococcus* lineages described here (Figure 7). Our evidence suggests that the ancient tetrapolar loci expanded to incorporate additional genes; this process began with acquisition of components of the pheromone signaling MAPK cascade *(STE20, STE11,* and*STE12)* into the ancestral pheromone/pheromone receptor locus (ancient class). This was followed by a second round of acquisition of genes with an unknown role in mating (intermediate class I). Next, the ancestral homeodomain locus acquired genes that we hypothesize function in the dikaryon or meiosis (*SPO14, RUM1;* intermediate class II) based on their known roles in S. cerevisiae and Ustilago maydis (Honigberg et al. 1992; Quadbeck-Seeger et al. 2000).

Subsequent chromosomal translocation fused the pheromone signaling and homeodomain cluster in one mating type, entrapping the set of most recently acquired species-specific genes (recent class). This created an intermediate tripolar mating stage in which one mating type had one large contiguous MAT locus and the partner retained the ancestral gene clusters formed from the unlinked tetrapolar loci. During this transitional tripolar intermediate stage, only half of the meiotic progeny would be fertile. The incipient alleles of the opposite mating type were then either gene converted onto the newly-formed bipolar chromosome or, more likely, were linked via dual recombination events (Figure 7), collapsing the tetrapolar system to a bipolar one via a transitional tripolar intermediate. Evolutionary pressure for this event enabling production of a higher proportion of fertile progeny would then have swept the population, fixing the linked bipolar α and **a** alleles and leading to extinction of the tetrapolar system. More recent inversions facilitated by repetitive sequence elements then produced more homogeneous bipolar structures resulting in suppression of recombination between MAT alleles. The high degree of identity shared by α and **a** alleles of the recent gene class suggests that the evolution of MAT is still occasionally punctuated by both inter- and intra-allelic recombination. Our model involving collapse of a tetrapolar system to a bipolar one is supported by studies of the basidiomycete *U. hordei;* this organism is closely related to the tetrapolar fungus *U. maydis,* but its tetrapolar loci have been linked and recombination suppressed between the two, resulting in the formation of a bipolar mating type system (Lee et al. 1999).

Thus, we hypothesize that a chromosomal translocation juxtaposed the two previously unlinked sex-determining regions and produced a bipolar mating type system in which the two distinct regions are linked by a common block of sequence information. Subsequently, inversions occurred that obscured these boundaries and, as a consequence, suppressed recombination between the two regions. What was the evolutionary pressure to suppress recombination between the two linked loci, leading to their rearrangement in not only one but all three lineages? If the ancestral tetrapolar system was not multiallelic, and the four mating types in the population were in equal proportions, then any given individual in the population could mate with only 25% of the other members. However, in a population in which the two loci are linked, any individual can mate with 50% of the other population members. A recombination event in the common spacer region would result in two new mating types in which *SXI1α* is now linked to the *STE3**a*** pheromone receptor gene, and in which *SXI2**a*** is linked to the *STE3α* pheromone receptor gene. These recombinants could mate with each other, but would not complete the sexual cycle with the original MAT**a** and MATα members of the population, because while cell-cell fusion would occur, two copies of only one of the homeodomain-encoding genes would be present, and both are required for completion of the sexual cycle (C. M. Hull, M. J. Boily, and JH, unpublished data). Thus, whereas the parental strains could mate with 50% of the population, the recombinants could not and would have a disadvantage. This selective pressure for fecundity could have driven the inversions that we hypothesize occurred to prevent recombination between the two linked sex-determining regions.

While we can reconstruct a likely model for the evolutionary events that drove the formation of the large MAT locus of *Cryptococcus,* the events that led to the initial formation of the homeodomain- and pheromone/pheromone receptor-based gene clusters involved in sexual processes are less clear. How can recombinationally suppressed sites such as the original tetrapolar loci initiate expansion to create a larger nonhomologous region? One likely hypothesis involves the presence of the large number of transposable elements in the genome. It has been suggested that the spread of a mobile element through a population can be facilitated by increasing the probability of sex in its host (Hickey 1982). Therefore, a transposon insertion adjacent to either the locus that controls initiation of the sexual stage (the pheromone/pheromone receptor locus) or its completion (the homeodomain locus), and that leads to a significant sexual advantage, would be subject to positive selection. These events, as linked to specific alleles of each locus, could therefore be expected to increase the size of the nonhomologous region. Furthermore, local transposition events have been shown to cause small rearrangements, including inversions, which would further expand the clusters (Daboussi 1997). These transposons may then have contributed to the original expansion of the locus, and would provide additional support for the proposed role of mobile elements in the evolution of sex (Hickey 1982).

Another striking parallel with the human Y chromosome is the coherence of genes with common functions. Eight of the ten most ancient genes encoded by or recruited to the fungal ancestral pheromone/pheromone receptor locus mediate pheromone production and sensing (see Figure 2), and the remaining two *(IKS1, MYO2)* may play related roles. Finally, Y-specific genes are maintained by intrachromosomal recombination and repair of genes embedded within palindromes in an inverted orientation (Rozen et al. 2003). This mirrors the *Cryptococcus* pheromone genes in a striking example of convergence to a common genomic configuration that ensures that genes required for fertility are preserved by intrachromosomal recombination in the absence of homologs on the opposite sex chromosome. While gene conversion has played an essential role in maintaining the multiple pheromone genes, it has also decorated the locus with multiple other examples that would provide no apparent fertility advantage.

An interesting feature in the evolutionary history of many genes encoded by sex chromosomes in mammals is the degeneration and loss of one functional copy, as has occurred dramatically on the mammalian X and Y chromosomes. This is in stark contrast with the fungal MAT locus, in which functional copies of each allele have been retained. The basis for this difference is that mammals are obligate diploids, and the haploid form occurs only as the gamete stage (sperm and egg) during the mammalian life cycle. By contrast, in fungi such as *C. neoformans,* the organism is viable as both a haploid and a diploid, and thus gene degeneration or loss of essential genes cannot occur in either sex-determining region, since the organism most commonly occurs as haploid cells in the environment. By comparing the gene composition of the **a** and α alleles of the *Cryptococcus* MAT locus, we see that each essential gene has been retained in both alleles, and that with the exception of the MAT-unique *SXI1α* and *SXI2**a*** genes, each other nonessential gene has also been maintained as a pair of alleles diverged to different degrees based on their date of acquisition to the locus and evolutionary constraints on sequence divergence. The nonessential genes are presumably maintained in a functional form because they serve a role in mating, and their loss would lead to sterile isolates that would be lost from the population, or they function in other roles that provide a survival benefit to the organism.

Another difference between the MAT locus alleles and the mammalian sex chromosomes is the size disparity between the X and Y chromosomes compared to a similar size in *Cryptococcus* for both the **a** and α MAT alleles, and therefore for their host chromosome. The MAT locus occupies 6% of the 1.8 MB chromosome on which it resides, and thus has not expanded to occupy nearly the entire chromosome, in contrast to the mammalian sex chromosomes. A more similar analogy to the fungal MAT locus is the sex chromosomes of the plant papaya *(Carica papaya),* in which the sex-determining region occupies only about 10% of the 41-MB primitive Y chromosome (Liu et al. 2004). In that example, the chromosomes are defined as sex chromosomes, and yet the sex-determining region has not yet expanded to capture the entire host chromosome on which it resides. Thus, there are two issues at play in determining the size of sex-determining regions: expansion, and gene degeneration and loss. Comparison of these divergent sex-determining systems in fungi, plants, and animals reveals both shared principles and unique features as these organisms have specialized to their particular environmental niche and survival strategy.

In summary, the *Cryptococcus* MAT locus resembles the structures hypothesized for the ancient human Y chromosome, in which recombination suppression was limited to a small portion of the chromosome around *SRY* (Skaletsky et al. 2003). Furthermore, this type of structure has been identified in the plant kingdom in studies that defined the sex chromosomes of the papaya (Liu et al. 2004). These parallels reveal that similar mechanisms drive the evolution of sex-determining regions in all three eukaryotic kingdoms, and establish *Cryptococcus* as a paradigm to elucidate molecular principles governing cell identity and sex chromosome dynamics.

## Materials and Methods

### 

#### Strains and media

The strains used for construction of bacterial artificial chromosome (BAC) libraries and analysis of the mating type alleles were C. gattii serotype B isolates WM276 (α) and E566 (**a**) from the Australian environment. E. coli DH5α was used as the library host strain. LB or FB media supplemented with the appropriate antibiotic was used for E. coli culture (Sambrook et al. 1989).

#### BAC and sequencing libraries

Large-insert libraries (insert sizes 100–120 kb) for the candidate C. gattii serotype B strains WM276 and E566 were constructed in pBACwich (Choi et al. 2000), a derivative of pBeloBAC11 (Cai et al. 1995), using HindIII partially digested genomic DNA (Lengeler et al. 2002). Clones from the BAC library were arrayed on nylon membranes (Sambrook et al. 1989) and hybridized to mating type-specific gene probes from *Cn.* var. *neoformans* to identify overlapping sequences that span the MAT locus (Lengeler et al. 2002). BAC DNA from two clones for each isolate (3K12 and 3O16 for WM276, and 3E18 and 1C03 for E566) was prepared using the NucleoBond BAC Maxi Kit (Clontech, Palo Alto, California, United States), and random insert libraries were constructed for each using randomly sheared 1.5- to 3-kb DNA fragments (GeneMachine HydroShear; Genomic Solutions, Ann Arbor, Michigan, United States) (Oefner et al. 1996) that were cloned into pUC18 (Yanisch-Perron et al. 1985). 1,100 clones were picked for each BAC random insert sequencing library.

#### Sequencing and assembly

Sequencing reactions were performed with an MJ Research (Reno, Nevada, United States) thermal cycler using standard BigDye chemistry (Applied Biosystems, Foster City, California, United States) and analyzed on an Applied Biosystems PE3700 96-capillary sequencer. Sequence reads were assembled using the PHRED/PHRAP/CONSED package (Ewing and Green 1998; Gordon et al. 1998). Additional analysis of the data was performed using BLAST (Altschul et al. 1990) and the GCG software suite (Wisconsin Package; Genetics Computer Group [GCG], Madison, Wisconsin, United States). Based on the initial assembly of the end sequences, oligonucleotides were selected to close gaps in the sequence coverage by primer walking.

#### MAT locus annotation

Genes were annotated in the C. gattii sequences based on homology to the existing annotation in C. neoformans; in some cases, this led to revision of the C. neoformans annotation. Additional genes that did not have previously defined homologs *(BSP1, BSP2, BSP3, GEF1,* and *NCM1)* were found by comparing the six available MAT allele sequences and by identifying large regions of identity that were unassigned to genes. Based on ClustalW v1.4 (Thompson et al. 1994) alignment and identification of intron consensus sequences, primers were designed and RT-PCR employed to characterize gene structures using total RNA and the Ready-To-Go RT-PCR Bead system (Amersham Biosciences, Piscataway, New Jersey, United States). RT-PCR products were directly sequenced using primer walking. Repeated elements in the *Cn.* var. *neoformans* locus were defined using the BLASTn algorithm to search the JEC21 genome generated at TIGR (www.tigr.org, 10/21/03 release).

#### Phylogenetic analysis

Protein-coding DNA sequences in FASTA format were automatically aligned using ClustalW 1.81 (Thompson et al. 1994). Each gene alignment was imported as a Nexus file into MacClade 4.05 (Maddison and Maddison 1997) and manually edited according to the superimposed amino acid sequences. Aligned data sets ranged in length from 130 to 5,600 bp. In each alignment, the start and the stop codon were excluded from the phylogenetic analysis, as were regions that could not be unambiguously aligned. Exhaustive searches under maximum parsimony and maximum likelihood criterion were conducted using PAUP* 4.0b10 (Swofford 1999) on each single gene data set. Model parameter estimates for the maximum likelihood analysis were obtained from Modeltest 3.06 (Posada and Crandall 1998). Statistical support was calculated using 1,000 bootstrap replicates under maximum parsimony and maximum likelihood. *K_s_* values for comparison of the **a** and α alleles of all MAT protein-coding genes in each lineage were calculated using DnaSP 3.51 (Rozas and Rozas 1999). The tree topologies of *STE20, ZNF1, SPO14,* and *RPO41* were compared with each other as representatives of the different strata by applying the Shimodaira-Hasegawa test (Shimodaira and Hasegawa 1999). Comparisons within a group were performed between two genes of the same stratum.

#### Genome-wide analysis of transposon content

To determine the relative frequency of transposons in the MAT locus alleles of *Cn.* var. *neoformans* compared to the rest of the genome, the locations of 35 previously identified transposable elements (Lengeler et al. 2002; Goodwin and Poulter 2001; Goodwin et al. 2003) were mapped on the TIGR JEC21 genome assembly. The relative transposon contents of the MAT and non-MAT regions were calculated as a percentage of total sequence occupied by both complete and partial transposons, yielding a 5.17-fold enrichment of transposons in the MATα allele and a 5.30-fold enrichment in MAT**a**. The locations of each element have been submitted to TIGR for inclusion in the imminent release of the *Cn.* var. *neoformans* serotype D genome paper. In this analysis the transposon-rich presumptive centromeric regions were excluded.

## Supporting Information

Figure S1 The Boundaries of the MAT Locus Are Sharply Defined by Loss of Sequence Identity The entire MAT**a** and MATα alleles plus an additional 10 kb flanking sequence from each species were subjected to a pairwise comparison using a window size of 100% identity over 30 bp. Sequence identity is indicated by dots, which join to form diagonal lines in regions of high sequence identity. Diagonal lines seen in the upper left and lower right corners represent the flanking sequences (greater than 99% identity) and those in the central portion correspond to those genes hypothesised to have entered the MAT locus most recently. The additional region of identity present in *Cn.* var. *neoformans* due to the fixation of the α alleles of the *NCM1*, *BSP3* and *IKS1* genes in the MAT**a** allele is circled in blue. Scale is given in kb.
(64 KB EPS).

Figure S2 Genes of the *Cryptococcus* MAT Locus Define Four Discrete Maximum Parsimony Phylogenetic Groupings Phylograms were generated for each protein-coding gene in MAT under the maximum parsimony criterion in an exhaustive search. Numbers next to branches indicate statistical support as calculated in 1,000 bootstrap replicates. Shaded trees indicate genes with an unusual phylogenetic pattern, due to either gene conversion or a hybrid phylogeny pattern.
(218 KB EPS).

Figure S3 Genes of the *Cryptococcus* MAT Locus Define Four Discrete Maximum Likelihood Phylogenetic Groupings The phylograms represent the single most likely trees for each protein-coding gene in the MAT locus. Trees were generated under the best-fitting evolutionary model in an exhaustive search. Numbers besides branches indicate statistical support as calculated in 1,000 maximum likelihood bootstrap replicates. Shaded trees indicate genes with an unusual phylogenetic pattern, due to either gene conversion or a hybrid phylogeny pattern.
(225 KB EPS).

### Accession Numbers

GenBank (http://www.ncbi.nlm.nih.gov/) accession numbers for the MAT locus alleles discussed in this paper are WM276 (AY10430) and E566 (AY10429). GenBank accession numbers for other genes discussed in this paper are *Cn.* var. *grubii* MAT**a** (AF542528), *Cn.* var. *grubii* MATα (AF542529), *Cn.* var. *neoformans* MAT**a** (AF542530), and *Cn.* var. *neoformans* MATα (AF542531).

## Abbreviations

BAC: bacterial artifical chromosome
MAPK: mitogen-activated protein kinase
MAT: mating type
mya: million years ago
TIGR: The Institute for Genomic Research
